# Modelling parametric uncertainty in large-scale stratigraphic simulations

**DOI:** 10.1038/s41598-022-27360-y

**Published:** 2023-01-16

**Authors:** A. Mahmudova, A. Civa, V. Caronni, S. E. Patani, P. Bozzoni, L. Bazzana, G. M. Porta

**Affiliations:** 1 Dipartimento di Ingegneria Civile ed Ambientale, Politecnico di Milano, Milan, Italy; 2 Eni SpA - Upstream and Technical Services, San Donato Milanese, Italy

**Keywords:** Applied mathematics, Statistics, Stratigraphy, Mathematics and computing

## Abstract

We combine forward stratigraphic models with a suite of uncertainty quantification and stochastic model calibration algorithms for the characterization of sedimentary successions in large scale systems. The analysis focuses on the information value provided by a probabilistic approach in the modelling of large-scale sedimentary basins. Stratigraphic forward models (SFMs) require a large number of input parameters usually affected by uncertainty. Thus, model calibration requires considerable time both in terms of human and computational resources, an issue currently limiting the applications of SFMs. Our work tackles this issue through the combination of sensitivity analysis, model reduction techniques and machine learning-based optimization algorithms. We first employ a two-step parameter screening procedure to identify relevant parameters and their assumed probability distributions. After selecting a restricted set of important parameters these are calibrated against available information, i.e., the depth of interpreted stratigraphic surfaces. Because of the large costs associated with SFM simulations, probability distributions of model parameters and outputs are obtained through a data driven reduced complexity model. Our study demonstrates the numerical approaches by considering a portion of the Porcupine Basin, Ireland. Results of the analysis are postprocessed to assess (i) the uncertainty and practical identifiability of model parameters given a set of observations, (ii) spatial distribution of lithologies. We analyse here the occurrences of sand bodies pinching against the continental slope, these systems likely resulting from gravity driven processes in deep sea environment.

## Introduction

Sedimentary basins are formed over hundreds of millions of years by the joint contribution of a number of physical, chemical and biological processes, such as erosion and sediment transport from source areas^1^, deposition over subsiding areas, differential subsidence due to tectonic activity^2^, deformations caused by the fluctuation of the relative sea level, compaction and lithification^3^. The evolution of sedimentary systems can be studied quantitatively by means of mathematical tools generally termed as stratigraphic forward models (SFMs). The first SFM, developed by Pitman in 1978, involves a synthetic stratigraphy and simulations included sea level variations as input parameters^1^. More recently, the capabilities of these models have been largely expanded and in recent studies SFMs have been used to characterize the evolution of complex sedimentary systems by predicting facies occurrence and distribution. The use of such numerical tools to simulate real systems proved useful to analyse multiple hypotheses and evaluate the role of contributing factors in controlling basin stratigraphy observed at present day^4–6^.

The key advantage of SFMs is that they address geological processes through a dedicated physically-based quantitative model, i.e. mathematical expressions which can be explicitly tied to the physical, chemical and biological drivers affecting the systems^7^ (e.g., sediment discharges, sediment transport coefficients, carbonate production rates). The output of a SFM is a simulated basin-filling sedimentary succession and a set of paleoenvironmental conditions, such as paleo-bathymetry and sediment properties distribution, over the spatial domain and simulation time interval^8^. This in turn leads to process understanding related to real sedimentary scenarios, e.g., to assess the impact of sedimentary sources^6^ location or tectonic scenarios^9^. However, recent SFMs commonly involve a considerable number of coupled physical and chemical processes, such that the number of input parameters to be specified can be in the order of 100–200. Some of these parameters can be constrained a priori through literature information, yet most of these quantities remain affected by a considerable uncertainty. This means that exploring the impact of each individual process by trial-and-error approaches becomes unfeasible. Similarly, highly parameterized models hinder the possibility to calibrate all the unknown parameters SFM model with the available proprietary data. In this context a trial-and-error manual calibration is extremely time consuming and leads to a single (deterministic) solution. Probabilistic approaches have been proposed to address these issues^10,11^. Recently a few studies^8,12^ presented automatic calibration procedures for the general purpose SFM Dionisos^13^. These studies typically analyse a restricted set number of uncertain input parameters when showing the results of sensitivity analysis and parameter calibration procedures. However, the selection of the set of calibration parameters and the joint consideration of their uncertainty is a crucial step in the application of SFMs in real cases. In this context a key element which is often not addressed is the assessment whether the available data allow a reliable estimation of model parameters, a procedure recently termed as identifiability analysis^14,15^.

The goal of this work is to demonstrate how a probabilistic approach can further enhance the capability of Stratigraphic Forward Models (SFMs) to allow efficient risk-assessment analysis^3,16^ while fully acknowledging the uncertainty which is inherent to process-based parameter values. We address these issues upon leveraging the method proposed by Patani et al.^7^, previously validated in a synthetic scenario and here applied and extended for the first time to handle a field case scenario.

The modelling strategy is applied and adapted to a real case scenario in the Porcupine Basin, located off the West coast of Ireland on the North-Atlantic shelf^17,18^. The selected test case presents uncertainties related to various processes contributing to sediment production and displacement, i.e., sediment source locations and dimensions, local baselevel, in situ carbonate production, transport and accommodation of sediments. Calibration data refer to the depth of interpreted seismic horizons, i.e., the depth of given geological surfaces (of given ages) with respect to the current sea level. On the contrary, the distribution of facies in the sedimentary space is unknown. A mapping of amplitude anomalies was performed on a set of 2D seismic lines crossing the basin with different orientations, showing a possible identification of sand bodies with reservoir-like properties. The obtained 3D forward modelling aims to verify the presence and identify the location of sand bodies pinching out against the continental slope. The main goal is to understand the geological settings of the selected area, to assess the spatial distribution of base-of-slope sands and of their suitability for exploration as a result of a process-driven, non-constrained forward simulation. Our method leads to predict locations of the expected reservoir-like sandstone bodies. The results are compared with qualitative indications obtained by seismic interpretations suggesting the possible locations of sand bodies.

From a methodological standpoint the key objective of this paper is then to show to how the proposed method can address these crucial points: (i) assess the fidelity of the model to the available data used for calibration, (ii) provide a probabilistic prediction for the location of sand bodies, (iii) explicitly quantify uncertainty in selected process-based model parameters, (iv) define the identifiability of the selected uncertain model parameters with the available data. The objectives are here pursued by starting from a full parameterization of the Porcupine basin test case where uncertainty is considered across whole set of potentially relevant sedimentary processes.

The paper is organized as follows. Section "Geological setup" includes a brief literature survey of the Porcupine basin sedimentary setting, while Section "Methods" illustrates the methodology employed. Section "Results" presents the results of the model calibration by discussing the statistical characterization of estimated input parameters and output quantities of interest. Concluding remarks end the work.

## Geological setup

**Figure 1:**
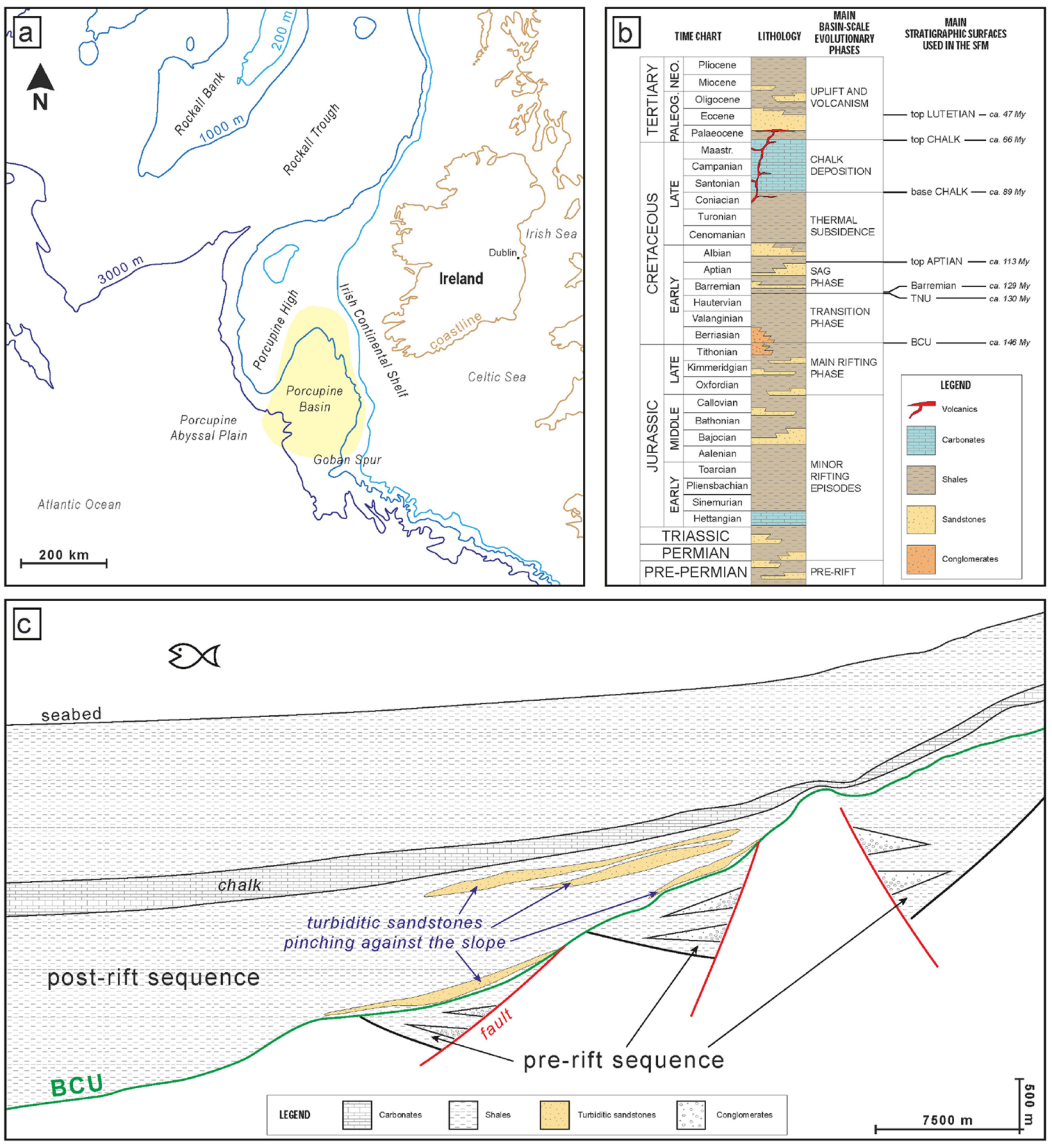
(**a**) Location of the Porcupine Basin (yellow) in the context of the Irish Atlantic margin. (**b**) Generalized stratigraphy of the Porcupine Basin (modified from Shannon, 1992^30^), with indication of the main tectono-sedimentary events. Names and ages of the eight calibrated depth surfaces deriving from 2D seismic interpretation are also shown (TNU stands for Top Neocomian Unconformity). (**c**) Qualitative idealized cross section across the eastern margin of the Porcupine Basin, derived from original seismic interpretations. The inferred position of updip-pinching base-of-slope turbiditic sandstones is shown. This kind of sandstones are the key object of the present study.

The Porcupine Basin is a large N-S oriented, sediment underfilled, offshore basin, located approximately 150 km to the west of Ireland. It is bordered to the north and west by the Porcupine High, to the east by the Irish western shelf, to the south by the Goban Spur, and to the west it opens to the Atlantic (Fig. 1a). The basin is approximately 300 km long and widens southwards up to a width of 200 km and it is considered an aborted early attempt of the northern Atlantic Ocean to open. Present-day water depths range from several hundred meters on the flanking shelves to more than 3000 m in the central and southern parts of the basin^19^. There is no evidence of oceanic crust at the basin floor except for the southern extremity^20,21^. The basin infilling history started during Jurassic and it is still developing^22,23^. The present-day basin configuration is the result of a geologic evolution started in Middle to Late Jurassic with a major rifting phase^25^, possibly continuing into the Early Cretaceous in a so-called “transition phase”^17,26,27^ (Fig. 1b). A transition to thermally-controlled subsidence accommodated a 4–6 km thick Cretaceous succession of largely deep-water sediments^24,28,29^ that draped and onlapped the earlier fault-controlled depocenters. Although the Early Cretaceous is generally considered a post-rift thermal subsidence phase during which deep-water conditions prevailed, the Lower Cretaceous succession contains numerous unconformities, some of which are associated with periods of increased clastic input^28^. Following the rifting phase, during which the basin is characterized by a dissected and rapidly evolving topography, in the Early Cretaceous subsidence became more uniform across the whole Porcupine, this evolutionary step being usually termed “sag” phase^21^.

Our study simulates the basin evolution from the Base Cretaceous Unconformity (BCU) to present day. A key objective is to characterize sediment distributions spanning from the BCU, which marks the end of the main Middle/Late Jurassic rifting phase, to the top of a regional scale chalk unit which dates lower Paleocene (Fig. 1b,c). The occurrence of possible clastic stratigraphic traps pinching against the southern border of the basin can be assumed from available seismic interpretations (Fig. 1c), but their location remains uncertain. Our aim is to better constrain the possible spatial location of such sandstone bodies (Fig. S1).

## Methods

### Overview and general workflow

**Figure 2:**
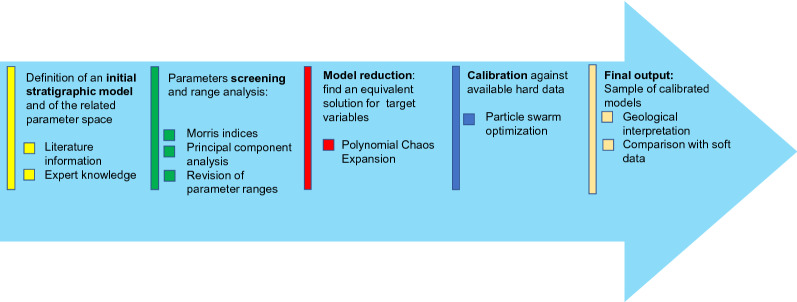
General scheme of the procedure.

Our numerical procedure is based on a series of four steps (see Fig. 2):

1. Definition of an initial geological model featuring a given model structure together with an initial guess of the model parameter variability ranges. Our model setup is here based on the Dionisos modelling framework^9,13^.
2. The screening step features a global sensitivity analysis whose aim is to diagnose the response of model outputs to uncertain input parameters based on the elementary effects method^31,32^. Then, a Principal Component Analysis (PCA) is performed to rank parameters importance^7,33^. As a result we discard parameters displaying a negligible influence on model outputs and we revise the initially defined parameters ranges.
3. Model reduction techniques are implemented to reduce the computational cost required to predict the key outputs of the problem. Surrogate models are typically constructed using data-driven approach via the response of the full simulator to selected locations in the parameter space^34,35^. Here, the surrogate model is built on the Polynomial Chaos Expansion (PCE) technique with a reduced number of parameters selected through the screening step^36–38^.
4. Stochastic inverse modelling is performed to estimate input parameters with available data. Here a Particle Swarm Optimization (PSO) technique is leveraged in a probabilistic framework^39–41^. Millions of parameter realizations are tested to select a sample of optimal parameters combinations, so the use of the surrogate model is important for computational efficiency.

The outcome of the procedure is a sample of model realizations, which, in turn, yields empirical probability distributions for both input parameter values considered optimal to fit the available data and output quantities of interest. The final results can then be compared with available geological interpretations and other soft data, similar to the ones presented in Fig. 1c. With respect to the method presented by Patani et al.^7^, we present a significant difference in the parameters screening step which is completed and reinforced by additional criteria and analysis (Section "Range analysis"), together with the probabilistic interpretation of model results in the context of risk-assessment analysis (see Section "Identification of geological features across the calibrated sample").

### Dionisos model

Dionisos is a process-based modelling tool designed to perform three-dimensional numerical simulations of basin-scale stratigraphic reconstructions^13^. Full model forward simulations were here performed with Dionisos, version 4.93. Dionisos requires specifying parameters related to physical, biological and geochemical processes including accommodation, production, erosion and transport of sediments. The simulations are performed in a user-defined time interval in a sequence of time steps. At each time step, three main tasks are performed:

- Definition of accommodation space for the sedimentation and its variation with time as a consequence of basin deformation due to eustasy, compaction or flexure;
- Definition of sediment supply which may correspond to a source or an in-situ marine carbonate production;
- Simulation of sediment transport using deterministic laws and the mass balance principle combined with the diffusion equation.

Transport of sediments is modelled by hillslope creeping and fluvial transport^9^. A planar two-dimensional sediment flux $Q_{sed,i}$ [km^2^/ky] ($i = X,Y$, being the two directions of space, see Fig. 2) is evaluated by these two mechanisms $Q_{sed,i} = Q_{HC,i} + Q_{FT,i}$, where

1 $$Q_{HC,i} = K_{s} S_{i}$$

2 $$Q_{FT,i} = K_{w} \left( {Q_{w} } \right)^{{N_{q} }} \left( {S_{i} } \right)^{{N_{s} }}$$

are sediment discharges generated by hillslope creeping and fluvial transport, respectively, ${K}_{s}$ and ${K}_{w}$ are constant slope- and water-driven diffusion coefficients [km^2^/ky], ${S}_{i}$ is the local topographical gradient along the $i$-direction, ${Q}_{w}$ is a dimensionless water flux, and ${N}_{q}$ and ${N}_{s}$ are fixed coefficients. Equations (1) and (2) are coupled with mass conservation to model the transport process^13^

3 $$\frac{\partial Z}{{\partial t}} = - \left( {\frac{{\partial Q_{sed,X} }}{\partial X} + \frac{{\partial Q_{sed,Y} }}{\partial Y}} \right)$$

where $Z$ [m] is the depth with respect to a fixed refence plane.

For the study case presented here the outputs of Dionisos are the depth maps associated with a given age and volume fraction of various types of sediments (sand, silt, shale, carbonate mud and carbonate grains) in the considered geological area, as described in Section "Dionisos model".

**Figure 3:**
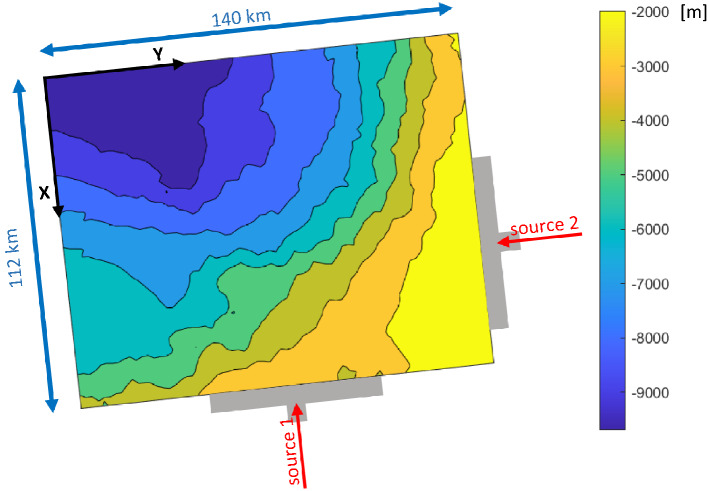
Planar geometry, bathymetry map at 146 Ma and location of the two sediment supply sources.

The considered geological domain consists of ${N}_{cells}=$ 56 × 70 = 3920 cells where the grid size is 2 km, thus covering 15,680 km^2^. The simulation considers the interval 146–0 Ma with a time step equal to 1 My. Two lateral sources of sediment supply are imposed in a stationary position and width located in the southern and in the eastern boundaries of the model. Figure 3 depicts the planar geometry together with the bathymetry map at 146 Ma (measured with respect to the current sea level) and the two sediment supply sources locations. Paleo-bathymetries are obtained from regional stratigraphic and sedimentological studies based on the seismic data interpretation.

### Uncertain inputs and output quantities of interest

We consider uncertainty in a set of 139 parameters grouped into four main categories, each corresponding to a specific process contributing to the stratigraphic simulation, i.e., (i) siliciclastic supply (68 parameters), (ii) carbonate production (3), (iii) transport and erosion (44), and (iv) eustasy and compaction (24). Siliciclastic supply from two sources (34 parameters in each) includes sediment and boundary fluvial discharges together with the volumetric fractions of sand, silt and shale. Carbonate mud production started at 90 Ma and was active until 66 Ma according to the expected basin stratigraphy (see Fig. 1b,c). Transport and erosion parameters are related to high and low energy events respectively i.e., for each type of sedimentary process, continental and marine coefficients associated with sediment lithologies (sand, shale, silt, carbonate grains and carbonate mud) have to be defined. Eustatic curve parameters represent the sea level at 11 specific ages (146, 145, 141, 140, 66, 65, 38, 37, 26, 25, 0 Ma) measured with respect to the current sea level. Finally, compaction parameters represent initial porosity, compaction law and residual porosity for the 5 sediment lithologies. For each of these quantities a reference interval of variability is defined based on expert knowledge and literature information. Parameters are then assumed to be stochastic variables described by a uniform probability distribution within the defined intervals. All the details related to the selected parameters ranges are provided as supplementary information.

Key model outputs are represented by the volumetric fractions of sediments (i.e., sand, shale, silt, carbonate grains and carbonate mud) and the vertical coordinate $Z$ [m] of the cells, measured with respect to the present-day sea level at each simulated age. Observations related to the depth maps are available and are collected in the vector ${\mathbf{Z}}^{*}$ [m]. This vector includes a vertical coordinate for each cell of the domain and for each selected age in the ages vector $\mathbf{a}=[146, 130, 129, 113, 89, 66, 47, 0]$ Ma, which correspond to the main evolutionary phase of the basin (see Fig. 1b). Our objective is to calibrate the Dionisos model using the data ${\mathbf{Z}}^{*}={Z}_{n}^{*}\left({a}_{j}\right), n=1:{N}_{cells}, j=1:{N}_{ages}$. This calibration is the used to infer distribution of sand bodies in the selected domain. Therefore, a key quantity that we will consider is the spatial distribution of the sand volumetric fraction within the computational domain. For a given cell $n$ we define the average sand fraction during the interval ${\Delta a}_{j}=[{a}_{j}, {a}_{j+1}]$ as ${\mathbf{V}}_{sand}={V}_{sand,n}\left({\Delta a}_{j}\right), n=1:{N}_{cells},j=1:{N}_{ages}-1.$ This quantity describes the average sand fraction for each cell and during each one of the seven time intervals ${\Delta a}_{j}$. There is no available direct measurement of the variable ${V}_{sand}$, but only qualitative information of possible accumulation areas in the domain (see Fig. 1c). Therefore, the spatial distribution of ${V}_{sand}$ is not used to calibrate model parameters but is computed as a predicted quantity of interest.

### Screening

Parameter screening is performed with a twofold aim: (i) identifying relevant parameters contributing to the selected goal (see Sections "Sensitivity analysis"-"Parameters selection") and (ii) evaluate the consistency between the assumed parameters intervals and the available data (see Section "Range analysis"). Both these steps are taken with a view to the parameter calibration step, to achieve a better identifiability of the calibrated parameters and at the same time to help maintaining a reasonable computational cost.

#### Sensitivity analysis

Sensitivity analysis is employed to rank parameters by quantifying their contribution to quantities of interest. We start here by considering a total number of parameters equal to $N$, where we set $N=139$ in the Porcupine test case, as described in Section "Dionisos model". To this end the Morris indices are employed^31^. These indicators are based on the evaluation of the elementary effect, ${EE}_{i}$, of the $i$-th parameter on a given output called $y$ in the following. Outputs of our model are spatial variables $Z({a}_{j})$ and ${V}_{sand}({\Delta a}_{j})$. The elementary effects are computed as

4 $$EE_{i} \left( {{\mathbf{p}}_{N} ,y} \right) = \frac{{\Delta y\left( {{\mathbf{p}}_{N} } \right)}}{\Delta } = \frac{{y\left( {p_{1} , \ldots ,p_{i} + \Delta , \ldots ,p_{N} } \right) - y\left( {{\mathbf{p}}_{N} } \right)}}{\Delta }$$

where $EE_{i}$ is the elementary effect associated with the variation of the $i$-th parameter, $\Delta$ is a given increment in the parameter space, ${\mathbf{p}}_{N}$ is the parameter vector and $y$ represents a scalar output corresponding either to ${Z}_{n}({a}_{j})$ or ${V}_{sand,n}({\Delta a}_{j})$ in a given cell of the domain. Elementary effects thus represent a discretization of the gradient of an output $y$ with respect to one parameter at a time, while other parameters are fixed.

We design $M$ trajectories across the parameter space where an appropriate value of $M$ can be chosen depending on the number of parameters^32^. The trajectories are built upon subdividing each selected parameter range in ${N}_{levels}$ which are equally spaced between the predefined minimum and maximum value of each parameter. The sampling is performed by changing a single parameter value between two subsequent points along a trajectory. Each node of the trajectory corresponds to a parameter realization for which a Dionisos simulation is performed thus providing the outputs of interest. Every trajectory allows then to evaluate an elementary effect for each parameter. After evaluation of the elementary effect ${EE}_{i,k}$ for each trajectory $k=1:M$ and for each parameter $p_{i} , i = 1:N$ , we consider the following indices:

5 $$\mu \left( {p_{i} ,y} \right) = \frac{1}{M}\mathop \sum \limits_{k = 1:M} EE_{i,k}$$

6 $$\sigma \left( {p_{i} ,y} \right) = \sqrt {\frac{1}{M}\mathop \sum \limits_{k = 1:M} \left( {EE_{i,k} - \mu \left( {p_{i} ,y} \right)} \right)^{2} }$$

7 $$\mu^{*} \left( {p_{i} ,y} \right) = \frac{1}{M}\mathop \sum \limits_{k = 1:M} \left| {EE_{i,k} } \right|$$

These indices represent the mean (Eq. (5)), the standard deviation (Eq. (6)) and the mean of the absolute values of $EE$s (Eq. (7)). These indices are calculated at each cell of the domain at every age in $\mathbf{a}$ for variables $Z$ and ${V}_{sand}$. The total number of simulations required by the procedure is ${N}_{real}=M\cdot (N+1)$.

#### Parameters selection

The screening procedure consists of two steps leading to successive reduction of the number of parameters. In the first step, we aim to discard the parameters that have negligible influence on the model outputs. To this end, for each age and output variable, we average the indices $\mu^{*}$ and $\sigma$ over the cells of the domain

8 $$\mu_{avg}^{*} \left( {p_{i} ,y} \right) = \frac{{\mathop \sum \nolimits_{n = 1}^{{N_{cells} }} \mu^{*} \left( {p_{i} ,y_{n} } \right)}}{{N_{cells} }}$$

9 $$\sigma_{avg} \left( {p_{i} ,y} \right) = \sqrt {\frac{{\mathop \sum \nolimits_{n = 1}^{{N_{cells} }} \sigma^{2} \left( {p_{i} ,y_{n} } \right)}}{{N_{cells} }} + Var\left( {\mu \left( {p_{i} ,y_{n} } \right)} \right)}$$

where $y_{n}$ refers to the output $Z\left( {a_{j} } \right)$ or $V_{sand} \left( {{\Delta }a_{j} } \right)$ in cell $n$. Quantity $\sigma_{avg}$ is calculated according to the law of total variance and $Var$ represents the variance of $\mu ({p}_{i},{y}_{n})$ over the cells of the domain.

We then define a reasonable threshold ${thr}_{Z}$ and ${thr}_{S}$ for $Z$ and ${V}_{sand}$, respectively. Then at every age in $\mathbf{a}$ we select the parameters for which ${\mu }_{avg}^{*}$ or ${\sigma }_{avg}$ is greater than these thresholds. We thus obtain two ${\mathbf{p}}_{j}^{Z}$ and ${\mathbf{p}}_{j}^{S}$ vectors containing the parameters having an impact for the simulation of $Z({a}_{j})$ and ${V}_{sand}(\Delta {a}_{j})$.

After selecting the important parameters for every model output, the first phase is finalized by taking the union of the sets of the important parameters for each output and for each age:

10 $${\mathbf{p}}_{sel} = \left( {\mathop \cup \limits_{j = 1}^{{N_{ages} }} {\mathbf{p}}_{j}^{Z} } \right) \cup \left( {\mathop \cup \limits_{j = 1}^{{N_{ages} - 1}} {\mathbf{p}}_{j}^{S} } \right)$$

The first step of the screening aims to neglect the parameters whose contribution is negligible on the whole set of outputs of interest, thus considering all the parameters contributing to the outputs in the next steps of the procedure. Vector ${\mathbf{p}}_{sel}$ will then be a vector of dimension ${N}_{sel}<N$.

The second step is performed with the reduced set of parameters ${\mathbf{p}}_{sel}$. Using this reduced parameter vector we repeat the sampling procedure introduced in Section "Sensitivity analysis" and thus obtain a new set of parameters realizations and the corresponding outputs evaluated via simulation through the Dionisos model. We then apply a Principal Component Analysis (PCA)^33^ to a matrix $\mathbf{J}$ whose components are the Morris indices $\mu$ defined in Eq. (5). More in detail we define the matrix ${\mathbf{J}}\; {\text{as}}$

11 $${\mathbf{J}} = \left[ {\begin{array}{*{20}c} {\mu \left( {p_{1} ,y_{1} } \right)} & \cdots & {\mu \left( {p_{{N_{sel} }} ,y_{1} } \right)} \\ \vdots & \ddots & \vdots \\ {\mu \left( {p_{1} ,y_{R} } \right)} & \cdots & {\mu \left( {p_{{N_{sel} }} ,y_{R} } \right)} \\ \end{array} } \right]$$

which gathers all the sensitivity indices for each parameter, cell locations and ages, and where $R$ is the number of individual output variable evaluations in each model output (for instance, for output $Z$, we consider a value for each cell for each simulated age, i.e., $R={N}_{ages}\times {N}_{cells}$). Matrix $\mathbf{J}$ can be considered as an approximation of the Jacobian matrix reporting the average value of the derivative of outputs with respect to input parameters. The contribution of each parameter to $Z$ and ${V}_{sand}$ is then evaluated based on the eigenvalues of the matrix ${\mathbf{J}}^{{\text{T}}} {\mathbf{J}}$ (for more details see^7^)

12 $$Contribution\left( {p_{i} } \right) = \frac{{\mathop \sum \nolimits_{k = 1}^{R} \mu^{2} \left( {p_{i} ,y_{k} } \right)}}{{\mathop \sum \nolimits_{j = 1}^{{N_{sel} }} \lambda_{j} }}$$

where $\lambda_{j}$ are the eigenvalues of the matrix ${\mathbf{J}}^{{\text{T}}} {\mathbf{J}}$. The first ${N}_{rdc}$ parameters according to this ranking are selected for $Z$ and ${V}_{sand}$, and we then consider the union of these two parameters set.

#### Range analysis

Parameter ranges are initially fixed a priori from the literature, geological proprietary measurements and expert opinion. To fix the range the values ${p}_{i,min},{p}_{i,max}$ are given. Considering the large number of parameters this task can be very challenging especially for complex geological domains. To assist the modelling, we propose here a quantitative method to assess the consistency between the selected parameter intervals and available observations.

To this end each parameter realization employed in the screening process is ranked according to the following objective function $J_{k}$

13 $$J_{k} \left( {{\mathbf{p}}_{k} } \right) = \mathop \sum \limits_{j = 1}^{{N_{ages} }} \sqrt {\frac{1}{{N_{cells} }}\mathop \sum \limits_{n = 1}^{{N_{cells} }} \left( {Z_{n} \left( {{\mathbf{p}}_{k} ,a_{j} } \right) - Z_{n}^{*} \left( {a_{j} } \right)} \right)^{2} } , \forall k = 1:N_{real}$$

where $Z_{n} \left( {{\mathbf{p}}_{k} ,a_{j} } \right)$ is the depth calculated by Dionisos simulation at cell $n$, age ${a}_{j}$ and for the $k$ th realization of the parameters ${\mathbf{p}}_{k}$. Smaller values of ${J}_{k}$ imply a smaller difference between data and model results. Thus, we perform the following steps:

1. Rank each parameter realization according to the value of ${J}_{k}$.
2. Select the realizations ${r}_{k}$ which lie in the $n$th percentile according to such ranking, thus obtaining the subset ${S}_{n}$.
3. Compute the probability distributions of a given realization being in the set ${S}_{n}$ for each selected level and for each parameter,
  14 $$F_{n} \left( {i,l} \right) = \Pr \left( {r_{k} \in S_{n} |p_{i} = p_{i} \left( l \right)} \right) \text{ with } i = 1:N_{sel} ;k = 1:N_{real} ;l = 1:N_{levels}$$

Quantity ${F}_{n}$ defines an empirical probability of finding a realization in the set ${S}_{n}$ conditional to a given parameter level, normalized by the absolute sample probability $\mathrm{Pr}({r}_{k}\in {S}_{n})$ (this latter being equal to $n/100$ by definition). The distribution of ${F}_{n}(i,l)$ across the levels is used to validate or revise the range for every parameter ${p}_{i}$. Notably, if the values attained by ${F}_{n}$ become large close to the interval boundaries this is interpreted as an indication that the fitness of the model may be increasing when the parameter approaches the value ${p}_{i,min}$ or ${p}_{i,max}$. These bounds are then revised to increase or reduce the width of the considered range. Specifically, in this study we have employed ${N}_{levels}=8$, thus we have analysed cumulative values of ${F}_{n}\left(i,l\right)$ at levels $l=\mathrm{1,2}$ and $l=\mathrm{7,8}$ to revise the lower and upper bound of the range of each parameter, respectively (see Section "Parameter screening results" for a numerical example). Note that different choices are possible for quantity ${N}_{levels}$. Increasing ${N}_{levels}$ is providing a finer discretization of the parameter range, but at the same time decreases the sample size of realizations available for a given level, thus increasing statistical noise in evaluating Eq. (14). In our preliminary test we found ${N}_{levels}=8$ is a reasonable compromise between resolution and computational cost. The outlined procedure aims at identifying macroscopic trends indicating that some specific values of a model parameter may lead to a clear departure from the observed data, while other values indicate a behaviour closer to the data. We recall that in this step of the procedure the sampling is not meant at optimizing the objective function reported in Eq. (13), therefore we cannot draw any conclusion about final calibrated parameters values and the idea is to merely correct any range that shows an important misalignment with the data and to help modelers to deal with large number of input parameters. Range analysis is performed in both steps of the screening procedure presented in Section "Parameters selection".

### Model reduction

Application of SFMs in the industrial context is hampered by the computational cost required for numerical simulations. Here, a surrogate model is employed to mimic the selected outputs of Dionisos model. The surrogate model is formulated through the generalized Polynomial Chaos Expansion (gPCE) technique which estimates the model outputs through a multi-dimensional polynomial formulation in terms of input parameters^42^. A given model output $Z\left( {\mathbf{p}} \right)$ (at every cell and age) is approximated as^37^

15 $$Z\left( {\mathbf{p}} \right) \approx Z_{PC} \left( {\mathbf{p}} \right) = \mathop \sum \limits_{j = 1}^{{O_{p} }} \alpha_{j} \psi_{j} \left( {\mathbf{p}} \right)$$

where ${\mathbf{p}}$ is the vector of $N_{rdc}$ uniformly distributed random input parameters, $\psi_{j}$ are orthonormal multivariate Legendre polynomials, and the number of polynomials terms $O_{p}$ is defined as

16 $$O_{p} = \left( {\begin{array}{*{20}c} {N_{rdc} + D} \\ D \\ \end{array} } \right) = \frac{{\left( {N_{rdc} + D} \right)!}}{{N_{rdc} !D!}}$$

where $D$ indicates the maximum degree of polynomial approximation with respect to a single parameter.

Evaluation of the gPCE coefficients, ${\alpha }_{j}$, entails solving the complete model to compute $Z\left(\mathbf{p}\right)$ for several combinations of the uncertain parameters. Coefficients ${\alpha }_{j}$ are computed through a least square minimization of the truncation error at sampling points. These latter are here assigned according to quasi-Monte Carlo selection^43^.

### Stochastic inverse modelling

Stochastic inverse modelling approach is based on the minimization of the objective function defined in Eq. (13), where the value of the depth is approximated upon employing the surrogate model approximation ${Z}_{PC}(\mathbf{p})$. The objective function $J$ is minimized through Particle Swarm Optimization (PSO) technique^39^ by changing the value of the parameters and thus obtaining multiple evaluations of the function ${Z}_{PC}(\mathbf{p})$. At the initial step of the procedure, $t={t}_{0}$, ${N}_{x}$ number of particles ${\mathbf{x}}_{j}$ are sampled randomly in the parameter space together with a random value of speed ${\mathbf{v}}_{j}$, $j=1:{N}_{x}$. These particles are then displaced in the parameter space to minimize the objective function. At each step, $t$, the objective function (Eq. (13)) is calculated for parameter combination corresponding to each particle ${\mathbf{x}}_{j}$. Particle positions and speeds are updated according to the following expressions^39,44^.

17 $${\mathbf{x}}_{j}^{t + 1} = {\mathbf{x}}_{j}^{t} + {\mathbf{v}}_{j}^{t} , { }\forall j = 1:N_{x}$$

18 $${\mathbf{v}}_{j}^{t + 1} = \omega {\mathbf{v}}_{j}^{t} + \varphi r^{t} \left( {{\mathbf{x}}_{best,j}^{t} - {\mathbf{x}}_{j}^{t} } \right) + \varphi r^{t} \left( {{\mathbf{g}}_{best}^{t} - {\mathbf{x}}_{j}^{t} } \right), { }\forall j = 1:N_{x}$$

where $\omega$ is the inertia weight, $\varphi$ is called cognitive coefficient and ${r}^{t}$ is a random coefficient updated at each step $t$. The vector ${\mathbf{x}}_{best,j}^{t}$ identifies the parameter combination providing the lowest objective function value among those experienced by particle $j$, while ${\mathbf{g}}_{best}^{t}$ is the location of maximum fitness (minimum distance to data) ever discovered by all particles of the swarm. Both these locations are updated at each iteration $t$. Convergence of the algorithm is obtained when an optimum of the objective function is attained or a predefined number of displacements is reached. In this analysis convergence criterion is set as a number of displacements which is equal to $T=1000$ which does not ensure the global minimum is attained but is a good compromise between the accuracy and the computational cost. At the final iteration we identify the best fit parameter value as $\widehat{\mathbf{p}}={\mathbf{g}}_{best}^{t=T}$. This procedure is repeated for different random initial parameters combinations, thus yielding a sample of ${N}_{calib}$ of parameter values ${\widehat{\mathbf{p}}}_{1},\dots {,\widehat{\mathbf{p}}}_{{N}_{calib}}$, each corresponding to the solution obtained minimizing the distance between the surrogate model and the data. This parameter sample is then used to simulate a sample of ${N}_{calib}$ realizations of the full stratigraphic model through Dionisos, thus obtaining the probabilistic characterization of all the outputs of interest for the selected parameter values.

## Results

### Parameter screening results

For the first step of the screening 50 trajectories are generated with 139 parameters resulting in 7000 Dionisos forward simulations. Sensitivity indices are calculated for ${Z}_{n}({a}_{j})$ depth and ${V}_{sand,n}({\Delta a}_{j})$ volume fraction at every age ${a}_{j}$ or interval ${\Delta a}_{j}$ and cell $n$ using Eqs. (8) and (9). Thresholds are defined for $Z$ and ${V}_{sand}$ as ${thr}_{Z}=5 m$ and ${thr}_{S}=1\mathrm{\%}$. These thresholds are considerably smaller than the range of variability of the model outputs and are below the accepted uncertainty in the outputs. Hence, at this step of the screening only the uninfluential parameters will be discarded.

**Figure 4:**
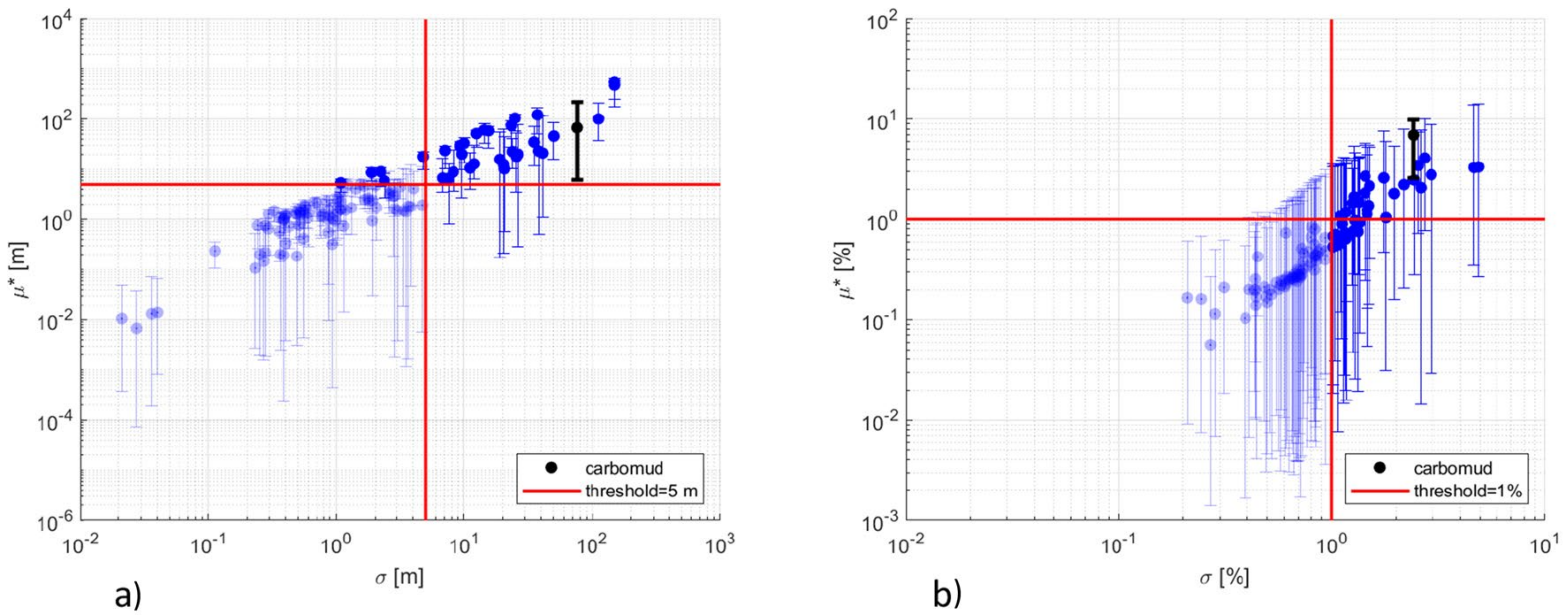
Sensitivity indices ${\mu }^{*}$ versus $\sigma$ at 89 Ma for $Z$ (**a**) and in the age interval 89–66 Ma for ${{V}}_{{sand}}$ (**b**). Red lines indicate the selected thresholds. Solid points are the spatial average values of ${\mu }^{*}$ and $\sigma$ for each of the model parameters, error bars indicate the 5th and 95th percentile of the index ${\mu }^{*}$ evaluated in the spatial domain. Results related to parameter #71 are highlighted in black. Parameters whose average indices are below the thresholds are shown half-transparent.

To exemplify the results obtained, Fig. 4 shows ${\mu }^{*}$ versus $\sigma$ at 89 Ma for $Z$ (a) and in the interval 89–66 Ma for ${V}_{sand}$ (b). Red lines represent the selected thresholds, while solid blue points represent the computed ${\mu }_{avg}^{*}$ and ${\sigma }_{avg}$ values. The parameters with ${\mu }_{avg}^{*}$ and ${\sigma }_{avg}$ falling in the lower-left quarter of the plots are deemed to be negligible for these specific outputs. For the outputs considered in Fig. 4, 105 and 103 parameters would be eliminated by considering separately $Z$ and ${V}_{sand}$, respectively. The error bars reported in Fig. 4 represent the variability of ${\mu }^{*}$ in the spatial domain by indicating range of values comprised between the 5th and 95th percentile.

**Figure 5:**
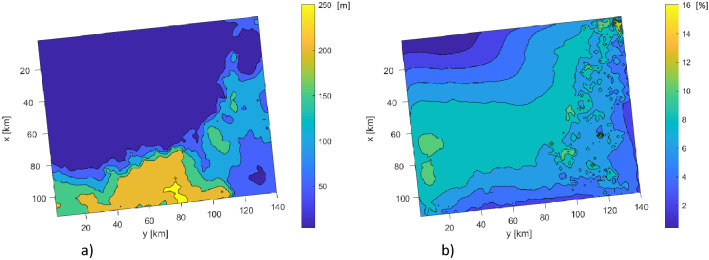
Spatial variability of ${\mu }^{*}$ for parameter #71 at 89 Ma for $Z$ (**a**) and in the age interval 89–66 Ma for ${{V}}_{{sand}}$ (**b**).

We exemplify the type of information that can be obtained from this analysis by studying the sensitivity of model outputs with respect to a single parameter related to carbonate production. The black point represents parameter #71. Figure 5 shows the spatial distribution of ${\mu }^{*}$ for this parameter at 89 Ma for $Z$ (a) and in the interval 89–66 Ma for ${V}_{sand}$ (b). Parameter #71 is carbonate mud production that is activated at 90 Ma in the chalk deposition phase (see Fig. 1b). Since parameter #71 activates carbonate muds accumulations at 90 Ma, it has negligible influence on ${V}_{sand}$ in the previous ages. However, this parameter can induce variations of the observed depth at 89 Ma up to 250 m on average in certain spatial locations, notably, in the southern region of the domain where carbonate deposits may accumulate in shallow marine environment (see Fig. 5a). Note also that the same parameter has a definite influence on ${V}_{sand}$ in the interval between 90 and 89 Ma. However, the spatial extent of the region where the parameter is important varies between the two outputs (compare Fig. 5a with Fig. 5b). In particular, the impact of carbonate production extends to a large portion of the domain when ${V}_{sand}$ is considered, with an important effect observed in the south-western corner of the domain.

**Figure 6:**
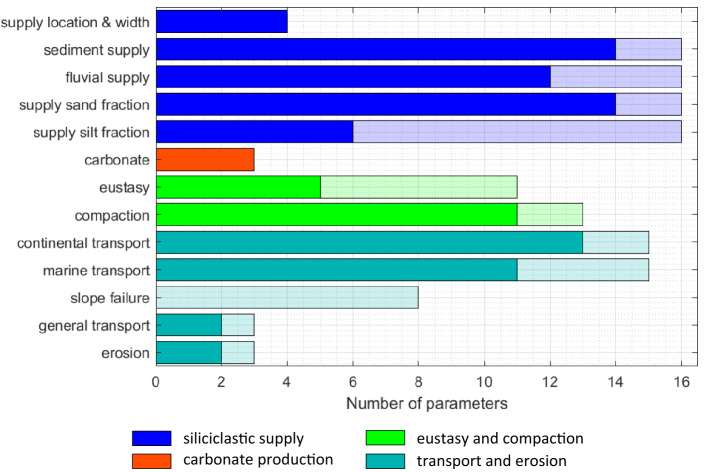
Distribution of the parameters selected in the first step of screening. Semi-transparent bars indicate initial number of parameters, solid bars indicate the number of parameters selected after the first screening step. For more details of the individual parameters found in each group see Supplementary information.

The final parameters set ${\mathbf{p}}_{sel}$ selected at this first screening step consists of 97 parameters neglecting 42 parameters out of 139. Figure 6 shows the distribution of the 97 selected parameters (solid bars) over all initial set (transparent bars) expressing different processes contributing to the overall sedimentological output. We observe that parameters associated with supply location and width and carbonate production are all considered important. On the other hand, slope failure parameters are always negligible, thus indicating such process is not contributing significantly to the selected outputs. Information like the one gathered here can assist modellers in identifying which processes and parameters are worth further investigation (for example through dedicated analyses of literature and proprietary data) and which can be conversely neglected.

**Figure 7:**
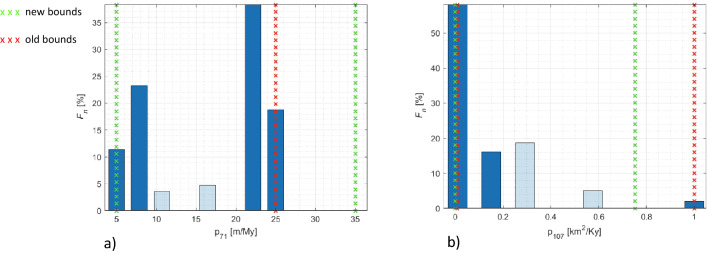
Distribution of the parameters #71(**a**) and #107 (**b**) delimited by red crosses together with the new intervals (green crosses) in the first step of the screening.

Following the procedure outlined in Section "Range analysis" we analyse the investigated parameters intervals. Figure 7 exemplifies two results of this procedure. Each bar in the plot represents the probability ${F}_{n}(i,l)$ to find a realization in the set ${S}_{n}$ at the considered level where $n=5$. Red crossed lines indicate the preliminary intervals while the green crossed lines correspond to the new intervals bounds. In Fig. 7a we observe that the distribution of parameter #71 (${F}_{n}(i=71,l)$, associated with maximum carbonate mud production at 90 Ma [m/My] is concentrated close to the right boundary. Hence, the parameter range is enlarged from the interval [5, 25] to [5, 35]. Figure 7b depicts the distribution ${F}_{n}(i=107,l)$, for parameter #107 which is slope driven marine silt transport coefficient [km^2^/Ky]. The total frequency ${F}_{n}$ is 2% in the two levels associated with the top 25% of the interval (corresponding with the last two levels). As a result, the procedure proposes a reduction of the upper bound of the interval from 1 to 0.75. It is important to note that range modification should be performed according to the physical and geological meaning of the parameters, therefore parameter modifications are finalized only after human intervention, i.e., after checking that the new proposed boundaries are not exceeding physical or geological meaning associated with given quantities (e.g., transport coefficients cannot assume negative values).

For the second screening step a new set of Dionisos forward simulations is performed with 97 parameters and new ranges. A total number of 4900 realizations are generated along 50 trajectories.

**Figure 8:**
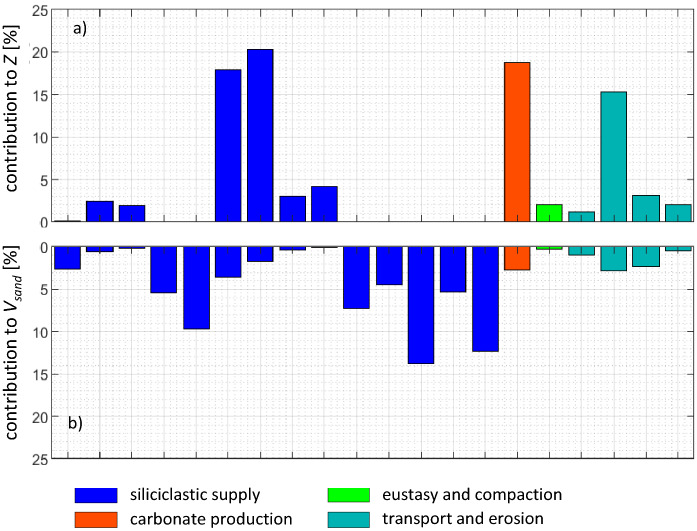
Parameter contributions estimated through Eq. (14) associated with the 20 selected parameters for $Z$ (**a**) and ${V}_{sand}$ (**b**). Numbers on the *x*-axis label indicate the parameter number, for their definition see Table 1.

Figure 8 shows the contribution defined in Eq. (12) of each selected parameter to the quantities of interest. We observe that the some of the selected parameters (#19, 20, 53, 54, 55, 58, 59) have negligible influence on $Z$, but are retained in the parameters list since they show a relevant impact on ${V}_{sand}$. Cumulative contribution of these 20 parameters to the $Z$ and ${V}_{sand}$ variability is 92.47% and 76.79%, respectively. This result is considered a good compromise in the model reduction process. The parameters selected at the end of the complete screening procedure are displayed in Table 1 together with their units and the final ranges of variability, obtained investigating parameter intervals as in the first screening step.

**Table 1:** Parameters used to build the surrogate model together with their units and final intervals.

Nr	Description	Unit	Final range
2	Sediment position source 1	km	40–85
6	Sediment supply source 1 at 113 Ma	km^3^/My	0–160
9	Sediment supply source 1 at 47 Ma	km^3^/My	0–150
19	Sand supply percentage source 1 at 146 Ma	–	5–20
20	Sand supply percentage source 1 at 130 Ma	–	5–20
37	Sediment supply source 2 at 146 Ma	km^3^/My	300–1050
39	Sediment supply source 2 at 129 Ma	km^3^/My	550–1300
42	Sediment supply source 2 at 66 Ma	km^3^/My	200–450
43	Sediment supply source 2 at 47 Ma	km^3^/My	400–550
53	Sand supply percentage source 2 at 146 Ma	–	5–15
54	Sand supply percentage source 2 at 130 Ma	–	5–12.5
55	Sand supply percentage source 2 at 129 Ma	–	5–15
58	Sand supply percentage source 2 at 66 Ma	–	5–15
59	Sand supply percentage source 2 at 47 Ma	–	5–15
71	Max compact carbonate production at 90 Ma	m/My	5–35
83	Initial silt porosity	–	33–37
97	High energy marine silt coefficient	km^2^/Ky	0.2–1.5
107	Slope driven marine silt coefficient	km^2^/Ky	0–0.5644
126	High-low water discharge ratio	–	0–30
139	Erosion basement thickness	m	0–30

### Identifiability of model parameters

We present here the results of model calibration, performed following the procedure outlined in Section "Stochastic inverse modelling". The calibration phase involves approximately 50 million evaluations. To perform the stochastic calibration the surrogate model is used as explained in Section "Model reduction". This enables the procedure to be computationally affordable compared to the Dionisos model, considering that a full model run requires more than one hour while the surrogate model is associated with a computational cost of about 1 s. The accuracy of the surrogate model in mimicking the full Dionisos simulations is discussed in supplementary materials.

**Figure 9:**
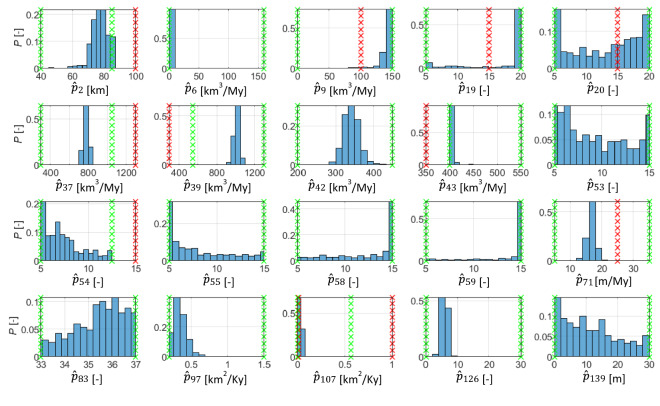
Histogram of the marginal probability distributions of input parameters obtained from the sample of 500 calibrations. Vertical red lines indicate the initial parameter range, green lines indicate the revised ranges obtained by following the procedure in Section "Range analysis".

Figure 9 depicts the marginal probability distributions of $\widehat{{p}_{i}}$ obtained from 500 calibrations. We can subdivide the parameters according to three groups according to the results:

- Identifiable parameters: this subset includes parameters whose marginal distributions are fully contained within the investigated ranges and display a single clear peak (such as parameters #2, 6, 37, 39, 42, 71, 97, 107, 126). For these parameters the procedure provides well identified best estimates and an approximation of their uncertainty. Estimates of parameters 6 and 107 are close to the interval boundaries, however they can also be considered identifiable, because the estimates are close to a physical limit for these parameters. For example, parameter 6 is estimated close to 0. This parameter physically corresponds to a solid discharge that cannot attain negative values, thus indicating that the parameter best estimate can be identified and is close to zero.
- Non-identifiable parameters: these parameters show approximately uniform distributions across the investigated range (see parameters #20, 53, 54, 83, 139 in Fig. 9). The procedure thus indicates that the estimation process is essentially insensitive to these quantities. It is important to note that these parameters are those exhibiting negligible contribution to $Z$, which is the quantity employed for calibration (see results in Fig. 8) and these results confirm the expected negligible influence on depth quantities. These results indicate that additional information is needed to identify these parameters, e.g., well data reporting an estimate of the volume fractions associated with various lithologies.
- Parameters requiring further investigation: some of the marginal distributions reported in Fig. 9 appear to concentrate near the boundary of the parameter intervals (# 9, 19, 43, 55, 58, 59). In general, such a result indicates that these parameters require further investigation, as their optimal range may be found outside the one initially selected for the estimation.

Close observation of the results given by the parameter estimation can yield interesting indications to interpret the geological setting in the Porcupine area. Generally, sediment inputs are predominantly entering the domain through the eastern source (source 2). However, a possible southern source (source 1) is included in the model. Estimates of the solid discharge of source 1 at 113 Ma (parameter 6) concentrate around 0, while the solid discharge at the same source is estimated close to the maximum allowed value of 150 km^3^/My at 47 Ma (parameter 9). This indicates the sediment delivery from the southern source (source 1, see Fig. 3) may have increased in recent ages while being relatively negligible at older ages. Note that the interval bounds originally assigned to parameters 6 and 9 favoured an opposite behaviour (larger values were allowed for parameter 6 if compared to parameter 9 in the initial ranges). Information gained from the parameter calibration process can then be used in relation to existing paleoclimatic, and paleogeographic models from which sediment supplies are usually assumed. Results could also be used in the context of so-called source-to-sink analysis to assess possible location and extent of past sediment sources and fairways. These parameters are typically plagued by considerable uncertainty and are related to larger spatial scales than the one considered to model a single sedimentary environment. Therefore, quantitative information like the one provided are relevant to future efforts aimed at improving the geological characterization of basin fills, such as the Porcupine basin’s one.

Figure 9 also reports updates of the parameter ranges resulting from the adopted criteria. These corrections mostly proved to be useful, although they did not avoid the occurrence of marginal distributions concentrating on the boundary of the intervals (see for example the distribution obtained for parameter 6). In most cases the reduction of the range led to the desired result, allowing to neglect portions of parameter intervals which are far from the optimal ones (see, e.g., parameter #37,39,107 in Fig. 9). Yet, in some cases reducing the parameter range appears unjustified in light of the results presented in Fig. 9. Neglecting portions of the parameter space may lead to an incomplete assessment of the parametric uncertainty, see e.g., the marginal distributions of parameters 2 and 43. This result suggests that modifications and particularly reduction of the considered ranges should be performed with care.

### Identification of geological features across the calibrated sample

The analysis of the seismic data collected from Porcupine basin shows the possible occurrence of sand bodies lying between the horizons corresponding at 129 and 113 Ma, i.e. the sag phase (see Fig. 1b,c). These sands are interpreted as being deposited in a submarine fan environment by subaqueous sediment gravity flows^45–47^. They could have been triggered by slope failures or by huge riverine floods entering sea water and funnelled along the slope as hyperpycnal flows^48,49^. Based on qualitative interpretations (see Fig. 1c) these deposits may show a pinch-out geometry towards the base of the continental slope, which is indicative of a possible stratigraphic trap that may hold hydrocarbons^50^. Pinch-outs are formed where the slope angle considerably decreases, allowing for sediment deposition. At seismic scale, vertical and lateral stacking of several depositional events generate large scale pinch-out geometries. During the assessment of the stratigraphic traps through forward modelling, it can be very challenging to locate the feeding channel, determine the accumulation potential of the sediments and detect the geometry of reservoir traps^49^.

**Figure 10:**
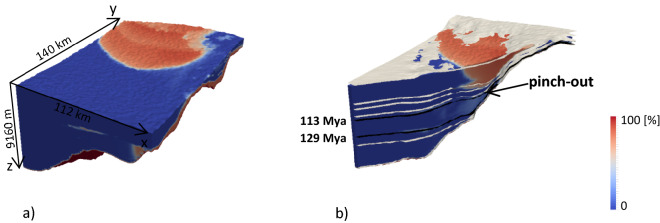
Three-dimensional representation of the exceedance probability $\mathrm{Pr}({\mathrm{V}}_{\mathrm{sand}}>20\mathrm{\%})$ evaluated over the sample of 500 calibrated solutions (**a**) and diagonal cross section of the same quantity together with the ${\mathbf{Z}}^{*}$ surfaces associated with available data in black and white (**b**).

Our aim is here to demonstrate how these features can be identified upon relying on our probabilistic approach. First, we consider the probability of observing sand accumulations, which can be expressed by the exceedance probability to observe a volumetric fraction of sand larger than a given threshold. Figure 10a depicts the exceedance probabilities $\mathrm{Pr}\left({V}_{sand}>0.2\right)$, which is based on the relative frequency of ${V}_{sand}$ predicted using the ${N}_{calib}$ realization of the full model. Figure 10b shows a diagonal cross section of the domain together with the seismic data ${\mathbf{Z}}^{*}$ surfaces drawn as white layers. Black layers represent the surfaces corresponding to the horizons corresponding at 129 and 113 Ma. The pinch-out is detected at the region where these two surfaces are close to each other. Figure 10b shows that the probability to find ${V}_{sand}>20\%$ in the pinch-out region is close to 20% over the 500 simulations.

**Figure 11:**
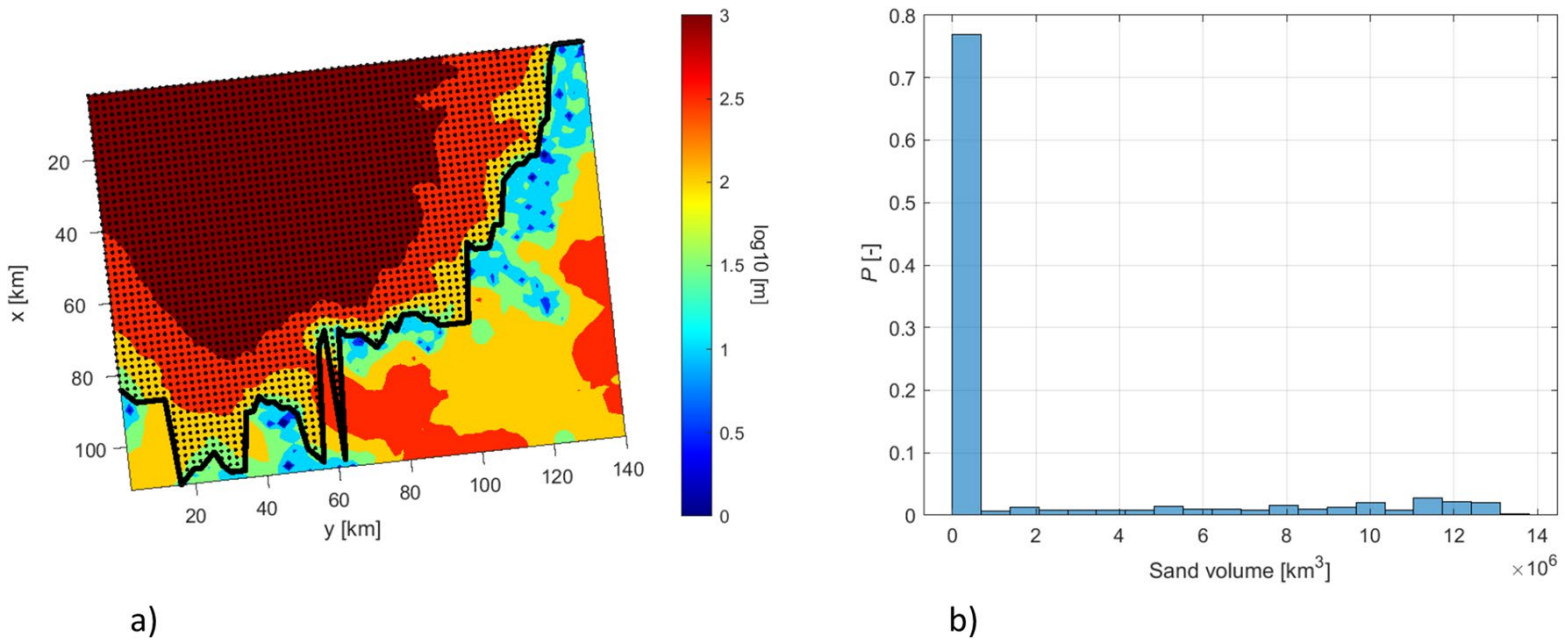
Spatial distribution of the sediment thickness $\Delta {\mathbf{Z}}_{113}^{129}$ together with the pinch-out location (black line), black dots identify the region of interest (**a**); marginal distribution of the cumulative volume of sand fraction in the region of interest (**b**).

To provide a more quantitative assessment of the sand accumulation downstream the pinch-out we have performed the analysis in two steps: (i) we identify a pinch-out location, by considering locations where the sediment thickness accumulated in the sag phase (between 129 and 113 Ma, $\Delta {\mathbf{Z}}_{113}^{129}$) becomes smaller than 50 m, (ii) we calculate the total volume of the sand in the region downstream (i.e., north-west with respect to the pinch-out line) for 500 simulations, the region of interest being indicated by black dots in Fig. 11a. Figure 11b shows that the accumulation of sand in this area is predicted only by 123 out of 500 simulations.

**Figure 12:**
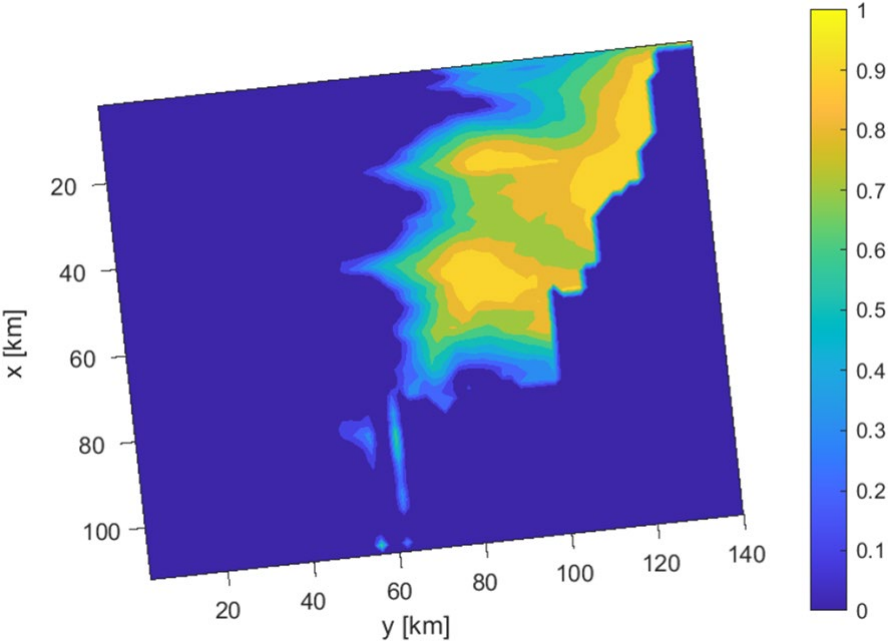
Spatial distribution of probability of observing ${V}_{sand}$>20% between 129 and 113 Ma in the selected 123 realizations.

With the aim of characterizing the geometry of these possible sand accumulations, we present the spatial distribution of the exceedance probability $\mathrm{Pr}\left({V}_{sand}>0.2\right)$ between 129 and 113 Ma in the restricted sample of the 123 realizations where sand accumulation is observed. Figure 12 identifies two sand bodies in the domain which can be interpreted as fan lobes. The probability map suggests that these two bodies can coexist as separate fans or together as one fan system. These results are significant in the context of exploration activities related to energy resources, e.g., exploration well placement, and to identify likely locations of stratigraphic traps for hydrocarbon accumulations. Our probabilistic approach can be used to propagate uncertainty to subsequent modelling steps such as petroleum system modelling. These latter typically require incorporating source locations and migration pathways and therefore need to consider an even larger scale than the one considered by stratigraphic models. Therefore, results such as the ones presented in Fig. 12 can be extremely useful to inform migration models on the local features of the system. In this context, our approach can provide probabilistic inputs for subsequent modelling analyses.

## Discussion and conclusions

We apply a probabilistic methodology to support quantitative geological interpretation through stratigraphic models in a real sedimentary setting. The Porcupine basin is considered as a test case for the application. The results obtained allow drawing general conclusions regarding the information value that can be gained from the application of the proposed method to a real scenario. Our study leads to the following conclusions:

1. Our procedure seamlessly integrates expert knowledge with reproducible quantitative indicators to reduce parameterization complexity of stratigraphic forward models. For the Porcupine basin case the uncertain parameter space is reduced from 139 to 20 dimensions, based on sensitivity metrics. The approach informs about the relevant contribution of individual processes and parameters to key targets of interests (e.g., horizon depths or sand accumulation). This screening step is complemented with a preliminary evaluation of the suitability of the selected parameter ranges in mimicking available observations. This information is then used to revise or corroborate expert opinion on the selected variability intervals for model parameters.
2. The probability distribution of calibrated parameters is obtained via an iterative procedure leveraging reduced order numerical model and a particle swarm optimization algorithm. We obtain an assessment of the practical identifiability of model parameters, this concept being associated with the possibility to reliably estimate parameters given a set of observations. Identifiability is assessed through a statistical postprocessing of the probability distributions associated with estimated parameters.
3. Our results lead to further insight on local sediment source dynamics. Sediment supplies in the Porcupine area are dominated by the eastern source whose discharge values are well identified by the proposed procedure. Our results indicate sediment inputs from southern sources are likely to be negligible at older times (113 Ma) while increasing to values exceeding the initial range at more recent ages (from 47 Ma).
4. Postprocessing of the results allows for identification of upcurrent pinching fan lobes in the so-called sag phase comprised between 129 and 113 Ma. We identify the location of sand accumulations, indicative of the occurrence of possible stratigraphic traps. This analysis provides a quantitative counterpart to qualitative interpretations (such as the one presented in Fig. 1). This analysis is conducted in a probabilistic framework where we identify and delineate volumes associated with sand fraction exceeding a given threshold. Probabilistic results can be readily used in the context of risk assessment procedures for subsurface resources exploration.

## Supplementary Information

Supplementary Information 1.

Supplementary Information 2.

## Data Availability

The data used to generate the results of this study are available upon reasonable request to the corresponding author.
