# Supplementary material for: Modelling parametric uncertainty in large-scale stratigraphic simulations
Source: Sci Rep. 2023 Jan 16;13:817. doi: 10.1038/s41598-022-27360-y (PMC9842771; doi:10.1038/s41598-022-27360-y)
Supplement: Supplementary file 1 — Supplementary Information 1. [file 41598_2022_27360_MOESM1_ESM.docx]

**Modelling parametric uncertainty in large-scale stratigraphic simulations**

**A. Mahmudova^1^, A. Civa^2^, V. Caronni^2^, S. E. Patani^1^, P. Bozzoni^2^, L. Bazzana^2^, G. M. Porta^1*^**

1 Dipartimento di Ingegneria Civile ed Ambientale, Politecnico di Milano, Italy

2 Eni SpA - Upstream and Technical Services, San Donato Milanese, Italy

*corresponding author: giovanni.porta@polimi.it

**Supplementary information S1**: validation of PCE results

Surrogate model is built with gPCE technique using the parameters reported in Table 1. As a training dataset a total of 17000 simulations are performed with forward Dionisos model. The generalized polynomial chaos expansion (gPCE) model is built to predict the elevation $Z$ as the output. The coefficients of gPCE are estimated through a k-fold cross-validation technique with 10 subsamples of 1700 realizations each. The accuracy of the model is calculated by RMSE between the Dionisos and the surrogate model:

${RMSE}_{n}(a_{j})=\sqrt{\frac{\sum_{i=1}^{N_{real}} \left( {Z_{n}(a_{j},\mathbf{p}_{i})}^{Dion}-{Z_{n}({a_{j},\mathbf{p}}_{i})}^{PC} \right)^{2}}{N_{real}}} \forall n=1:N_{cells}, \forall j=1:N_{ages}$ (S1)

With 17000 simulations the best approximation (smallest $RMSE$ values) is obtained with a PCE model built with the polynomial order $D=3$. Fig. S1 shows the results of the prediction through PCE technique. Plots on the left are the bivariate histograms of $\mathbf{Z}$ elevation PCE prediction with polynomial degree 3 versus Dionisos model, and the colour maps on the left are the spatial distribution of $RMSE$ for all the ages 0, 47, 66, 89, 113, 129, 130, 146 Mya labelled as (a)-(h), respectively. We observe that in all the ages the south-east corner of the domain has higher values of $RMSE$. It can be explained by the fact that this part of the domain has higher gradients of the ground elevation and moreover, the two lateral sources from the south and the east both contribute to sediment fluxes in this region. Hence, it is challenging to build an accurate predictive model in the south-east region. However, the maximum value of $RMSE$ over all the ages and all the cells is 67.95 m. Considering the scale of the domain and the variability of $Z$ this is an acceptable inaccuracy. Table S1 lists the minimum, maximum and the average values of the spatial $RMSE$ for all ages in $\mathbf{a}$.


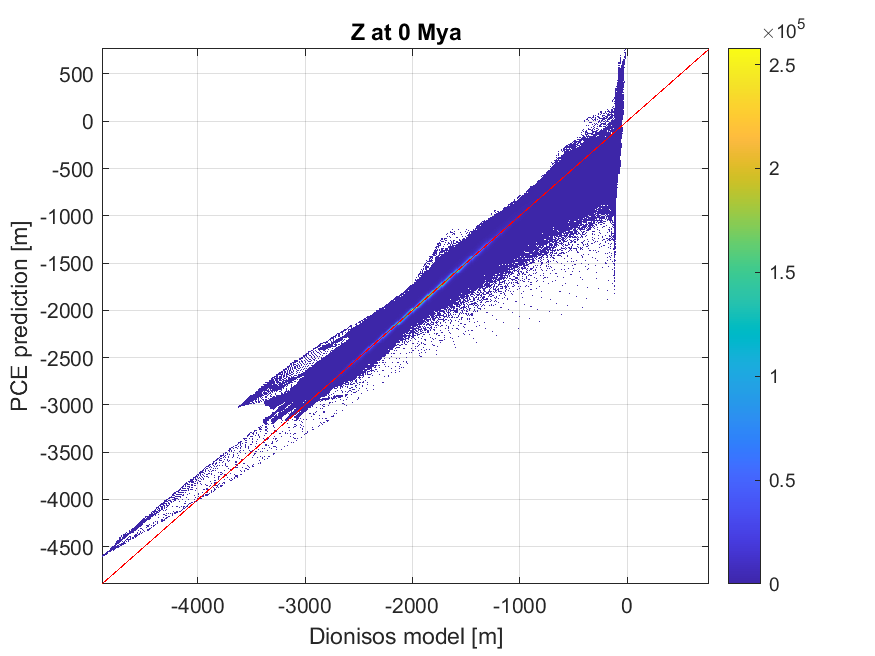

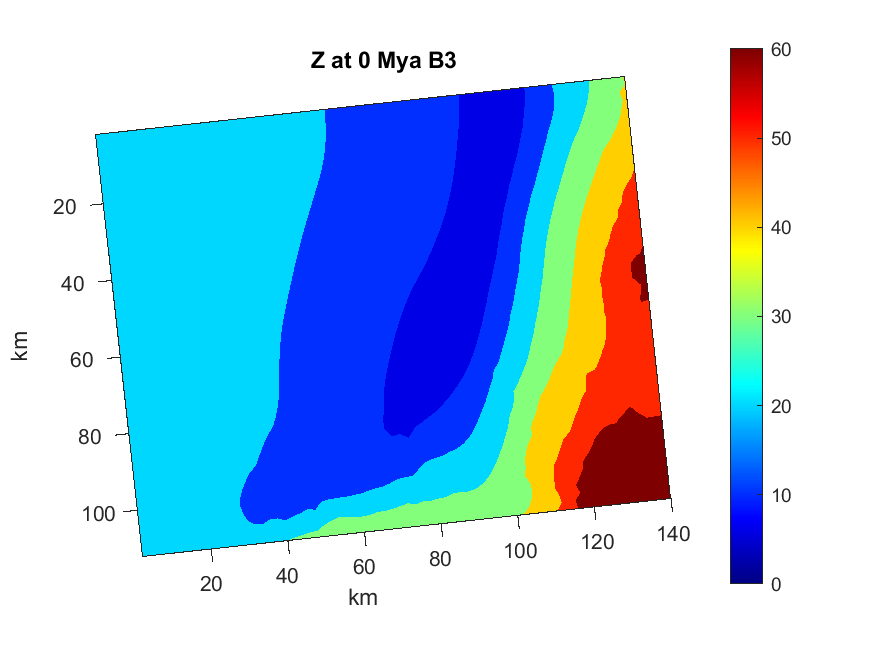


*a)*

[m]

[-]


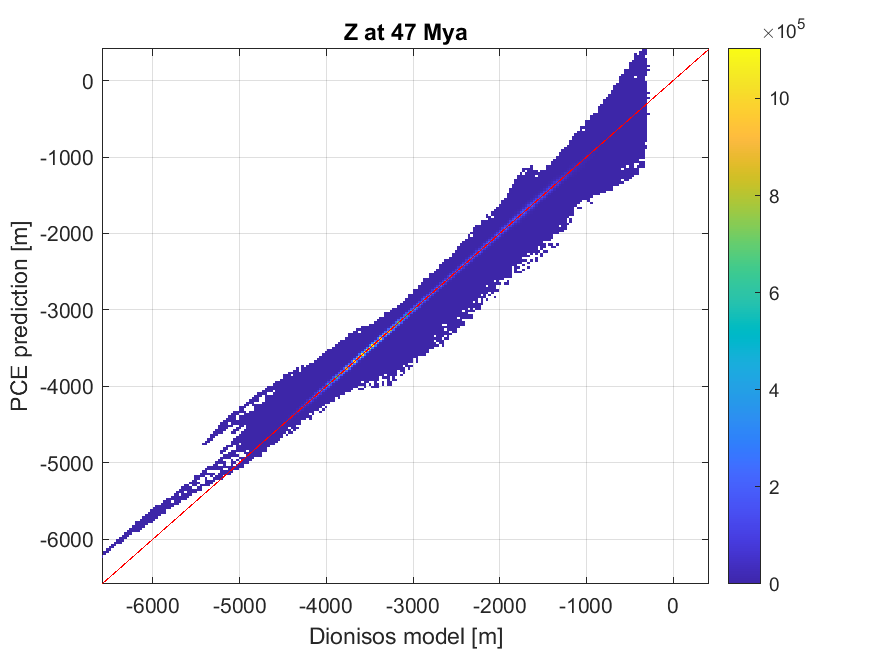

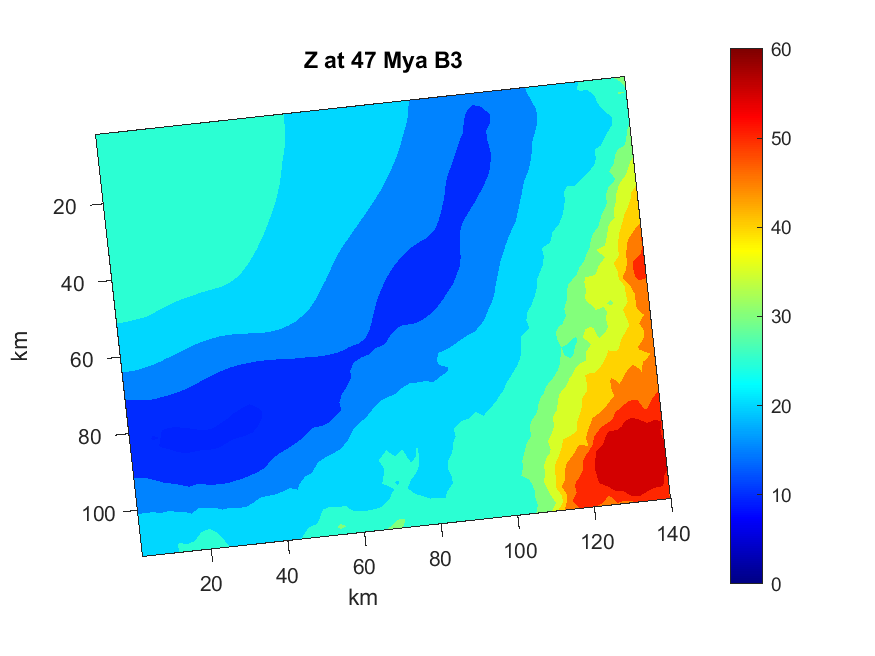


*b)*

[m]

[-]


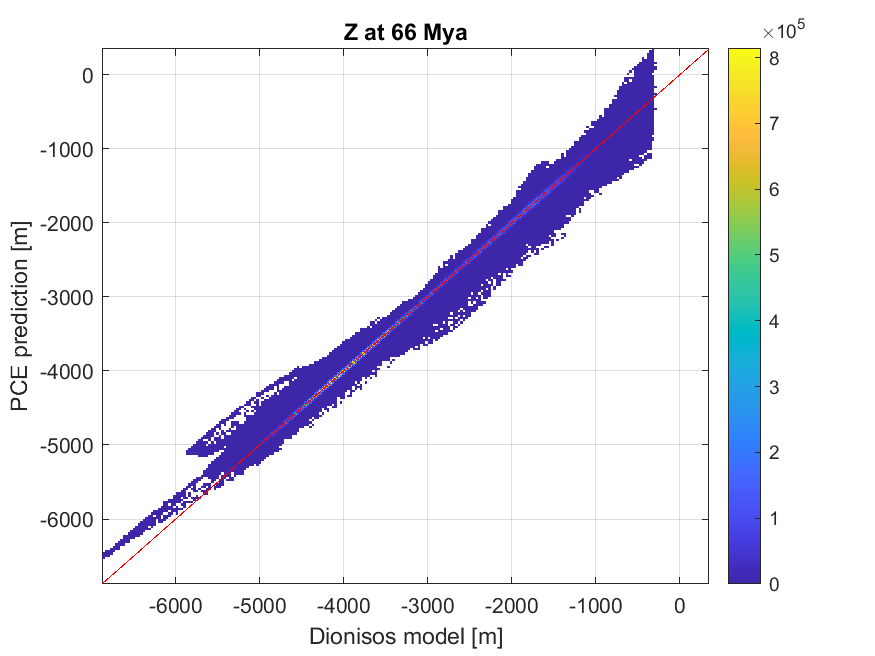

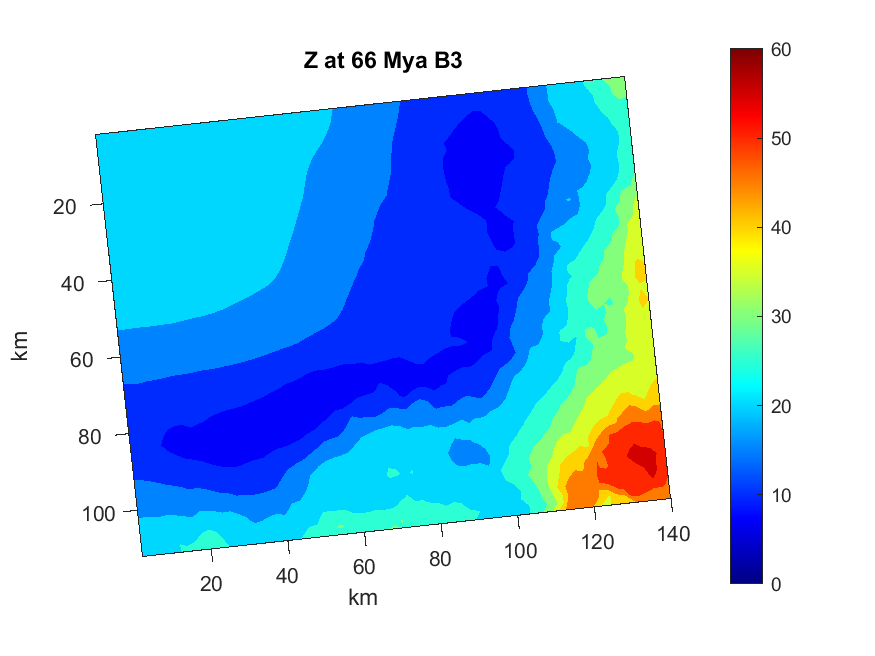


*c)*

[m]

[-]


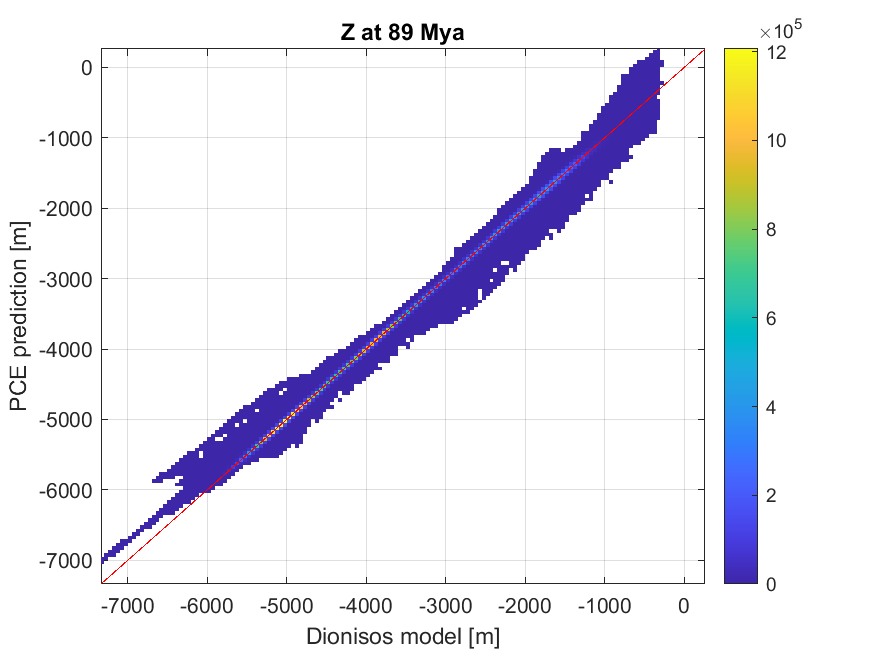

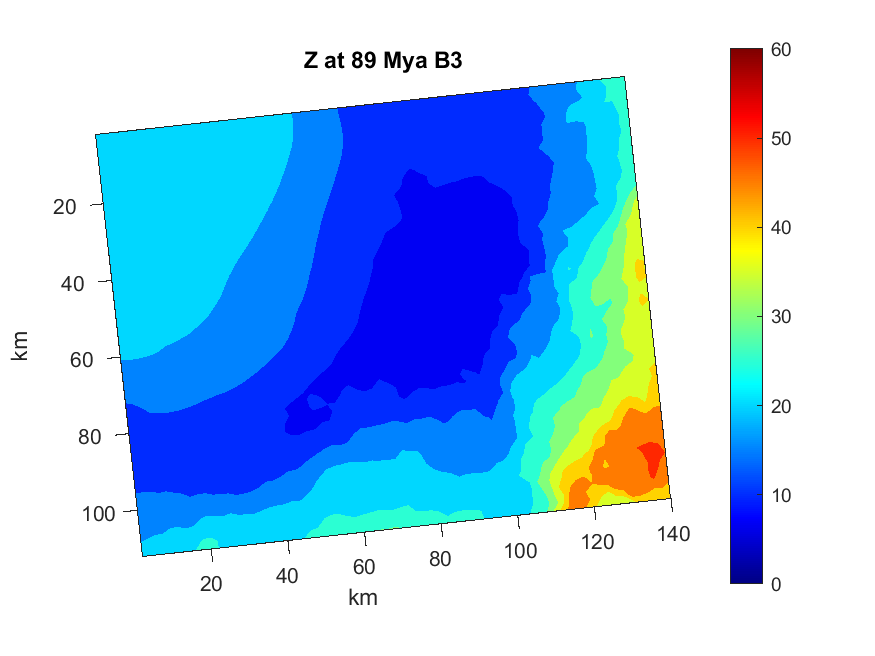


*d)*

[m]

[-]


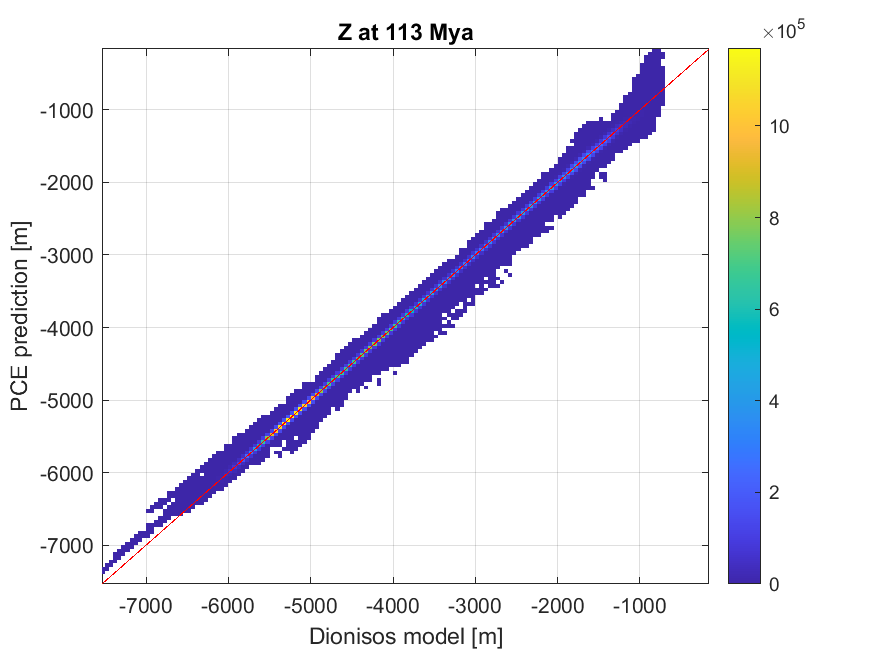

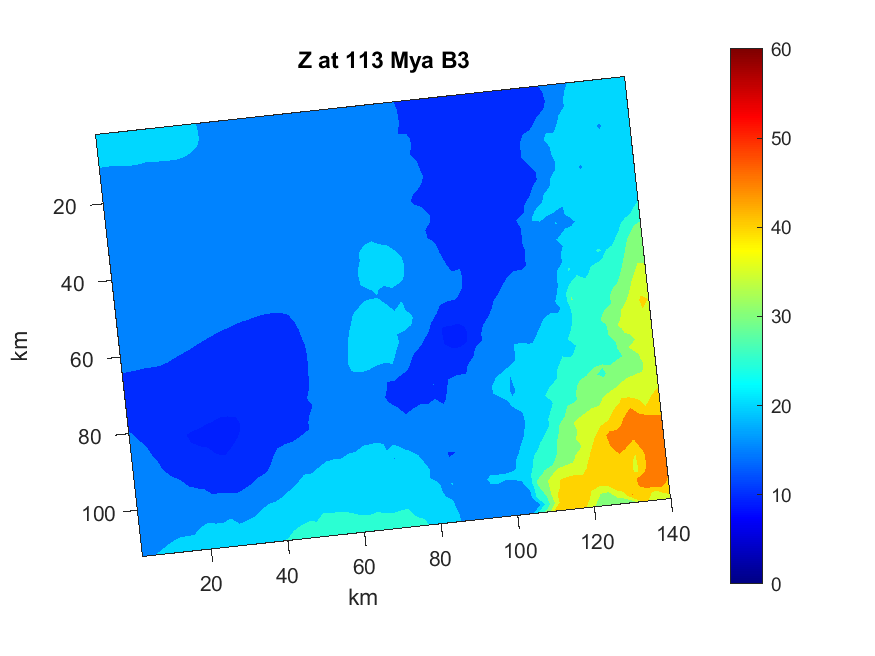


*e)*

[m]

[-]


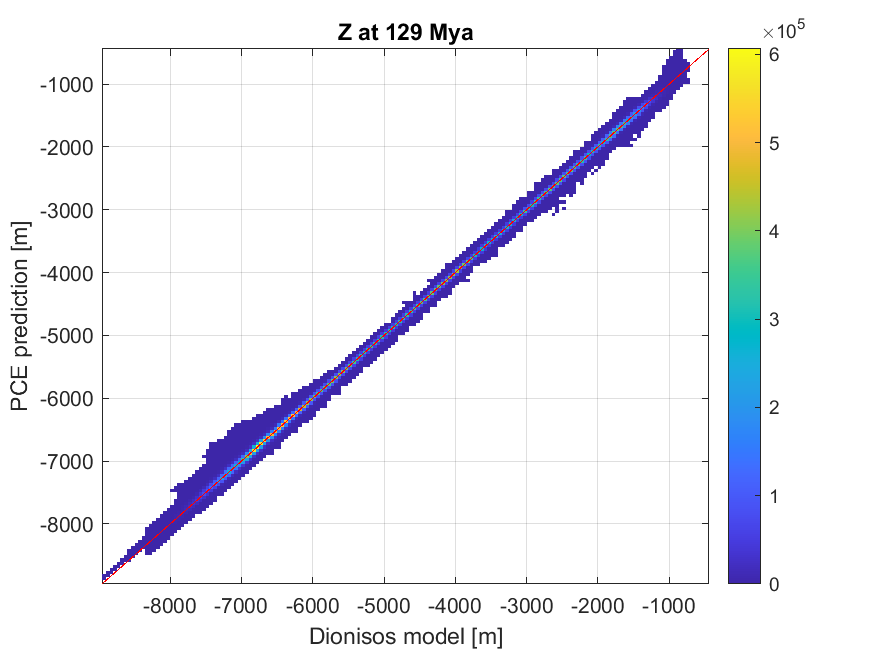

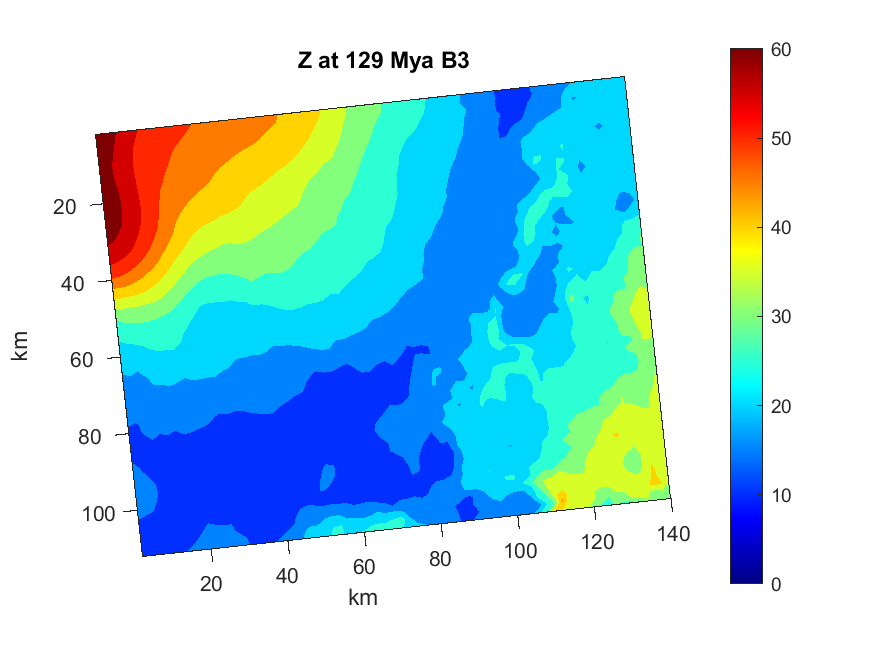


*f)*

[m]

[-]


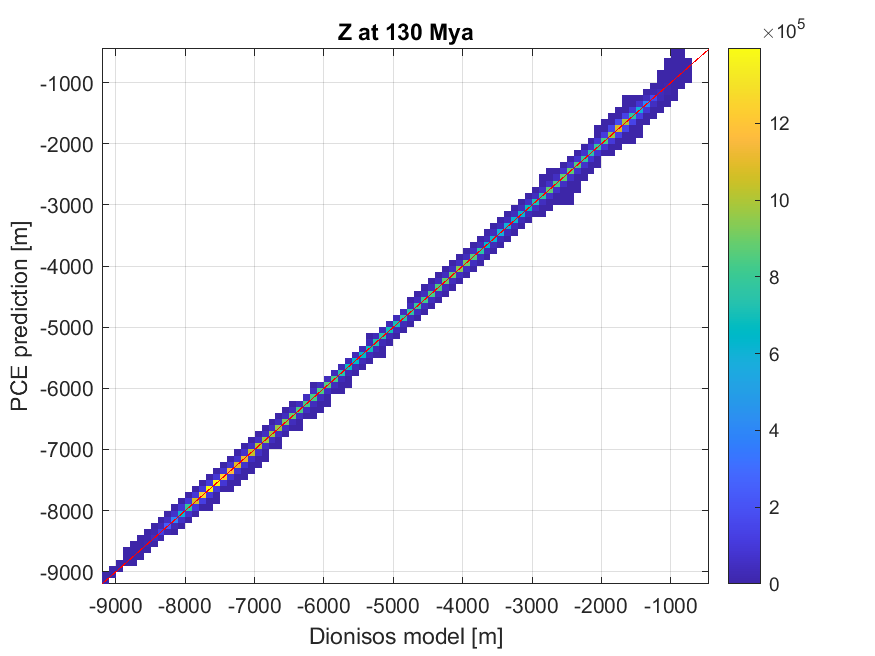

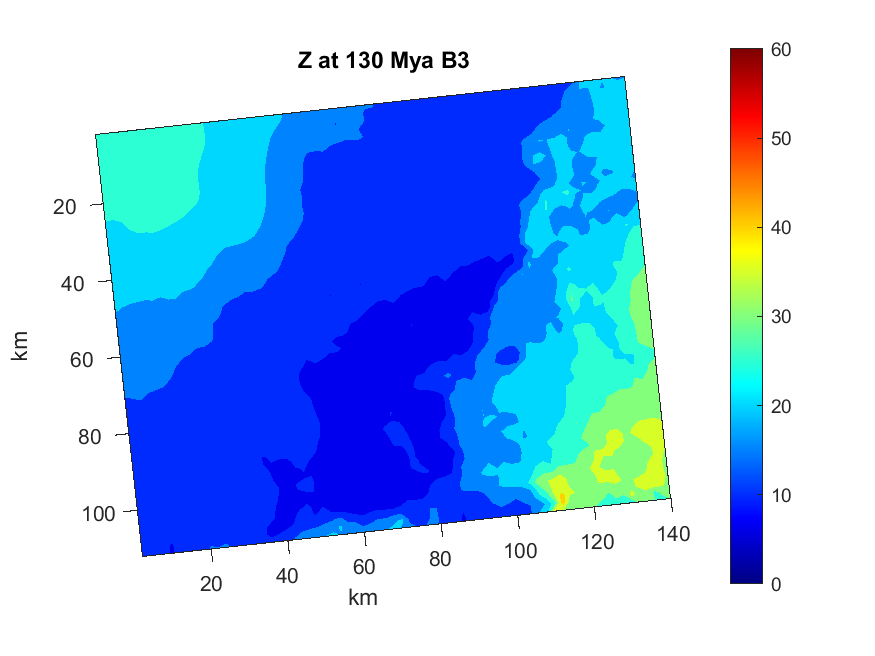


*g)*

[m]

[-]


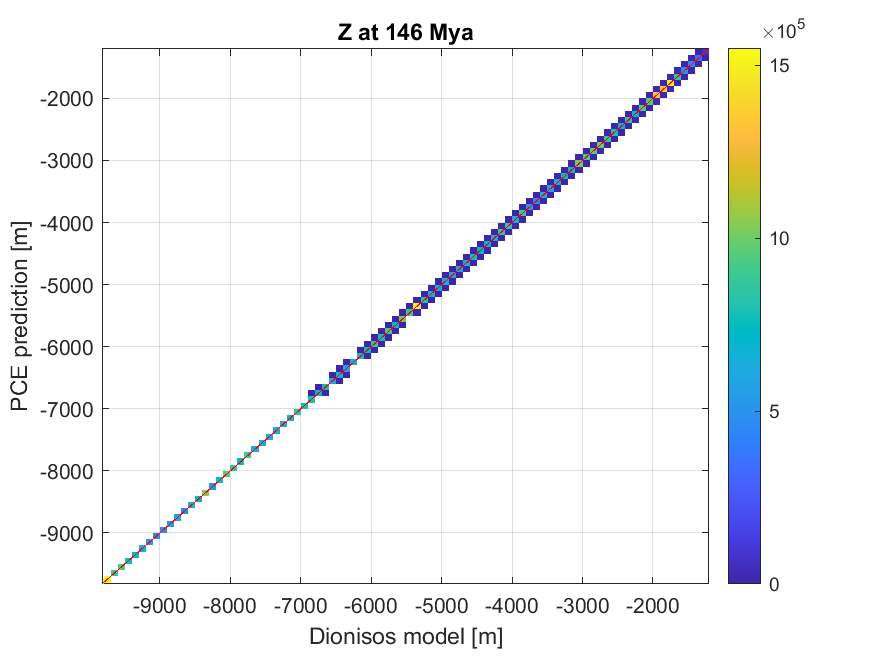

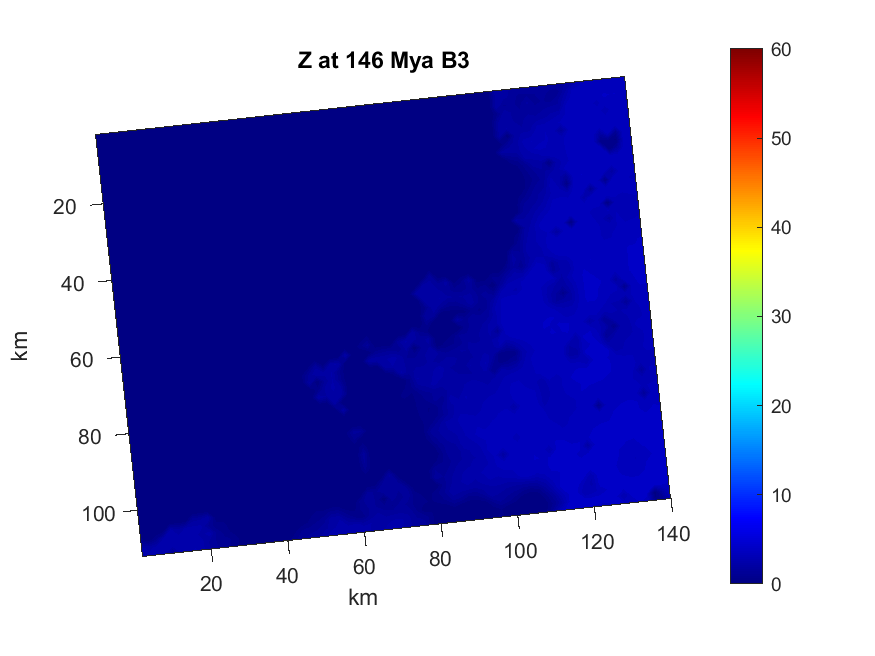


*h)*

[m]

[-]

Figure S1: Bivariate histogram of gPCE prediction versus the Dionisos model (left) and the spatial distribution of $RMSE$ (right) at ages 0 (a), 47 (b), 66 (c), 89 (d), 113 (e), 129 (f), 130 (g), 146 (h) Mya.

| **Age (Mya)** | **Minimum** $\boldsymbol{RMSE}$ **[m]** | **Maximum** $\boldsymbol{RMSE}$ **[m]** | **Average** $\boldsymbol{RMSE}$ **[m]** |
| --- | --- | --- | --- |
| 0 | 6.00 | 67.95 | 25.30 |
| 47 | 9.37 | 58.95 | 24.28 |
| 66 | 7.02 | 56.25 | 20.17 |
| 89 | 6.68 | 51.42 | 19.10 |
| 113 | 9.24 | 48.23 | 19.67 |
| 129 | 10.24 | 63.97 | 25.12 |
| 130 | 6.33 | 43.48 | 16.73 |
| 146 | 0 | 4.25 | 1.16 |

Table S1: Minimum, maximum and average values of the spatial $RMSE$ for all the ages in $\boldsymbol{a}$
